# Improving Pediatric Patients’ Magnetic Resonance Imaging Experience With an In-Bore Solution: Design and Usability Study

**DOI:** 10.2196/55720

**Published:** 2025-02-13

**Authors:** Annerieke Heuvelink, Privender Saini, Özgür Taşar, Sanne Nauts

**Affiliations:** 1 Imaging Innovation Philips Medical Systems Eindhoven Netherlands; 2 Data Science and AI Royal Philips Eindhoven Netherlands; 3 Ambient Experience Philips Medical Systems Best Netherlands

**Keywords:** MRI, magnetic resonance imaging, imaging, radiology, pediatrics, children, patient guidance, patient experience, design, usability, breath hold

## Abstract

**Background:**

Annually, millions of children undergo a magnetic resonance imaging (MRI) examination. Hospitals increasingly aim to scan young children awake, as doing so benefits both patients and health care systems. To help hospitals reduce the need for anesthesia, we have developed solutions to prepare pediatric patients at home and in the hospital.

**Objective:**

The goal of our project was to design, develop, and test a solution that extends our preparation solutions by guiding and engaging children during their MRI examination.

**Methods:**

Pediatric In-bore was designed to deliver a familiar experience by reusing design elements from our preparation solutions. It offers child-friendly movies and auditory and visual guidance about examination progress and breath holding. To evaluate children’s liking and understanding of the solution, we conducted a usability study. Ten healthy children participated in a mock MRI examination featuring pediatric In-bore. We observed task compliance (ability to lie still and hold one’s breath) and conducted guided interviews to assess their experience and understanding of the guidance offered.

**Results:**

Participants (aged 5 to 10 years) were generally positive about pediatric In-bore. They liked the main character (Ollie the elephant) and her movie. Auditory and visual guidance were generally liked and understood. All but one participant successfully managed to lie still during the mock examination, and 6 (60%) out of 10 participants successfully held their breath.

**Conclusions:**

Pediatric In-bore appears promising for engaging and guiding young children during awake MRI. It completes the Pediatric Coaching solution that now offers guidance throughout the MRI journey. Future research can expand on this work by evaluating the clinical impact of the Pediatric Coaching solution in a larger and more diverse sample of pediatric patients.

## Introduction

### Background

Using magnetic resonance imaging (MRI), physicians can diagnose a host of different pediatric conditions without exposing children to harmful ionizing radiation. Every year, millions of children get an MRI examination. To be able to have a successful examination, children need to enter a room with a large machine, lie down on a table that slides into this machine, and keep very still for an extended period of time (20-40 min, with some examinations taking up to an hour [1]). For some examinations, children need to hold their breath during various scans to enable physicians to create sharp images of the child’s heart or abdomen. During the examination, the MRI scanner makes very loud noises. Children report that this noise and boredom are the most stressful factors of having an MRI [2].

Given these challenges, young children are often scanned using general anesthesia or sedation, which enables high-quality scans along with a smooth and predictable workflow. Unfortunately, using anesthesia or sedation is costly, and waiting lists are typically longer for examinations with anesthesia or sedation than for awake examinations [3]. More importantly, there are concerns about the potential effect of repeated anesthesia exposure on children’s neurodevelopment (for an overview, see [4-7]). For these reasons, an increasing number of hospitals are trying to scan children awake.

### Existing Solutions

There are many initiatives to support hospitals that aim to scan children awake (see [7] for a review). Some initiatives focus on preparing children at home, for example, using a gamified app or virtual reality (eg, [8-13]). Other initiatives focus on preparing children in the hospital, for example, using face-to-face training programs with child life specialists [14,15] or educational (mock) scanners (eg, [16], for a review see [17]). During the MRI examination, children can sometimes watch a movie in the bore of the MRI scanner (eg, using movie goggles or a screen), play a digital game [18,19], or enjoy a child-friendly imaging room (for a review of different approaches, see [20]).

Next to initiatives that focus on one specific touchpoint in a child’s diagnostic pathway (such as interventions at home or in the hospital or the MRI room), there are multifaceted concepts that cover the entire pathway from home to examination. An example of such a holistic approach is Children Centered Care [21], which prepares children at home (with an app) and in the hospital (using an educational scanner) and supports them during their MRI examination with a child-friendly imaging room. The Pingunauten program [7,8,18] prepares children at home but also provides an in-bore game featuring the same child-friendly characters and theme (penguins in space) as the Pingunauten app.

To help hospitals reduce the need for anesthesia or sedation, we have previously created a gamified mobile app to prepare children at home (Scan Buddy; [22]). We also redesigned an educational scanner for the waiting room (the Kitten Scanner; [23]) to feature the same design elements, voice, and terminology as Scan Buddy. The main character featured in our solutions is a child-friendly elephant that is well-liked by children: Ollie the Elephant [24]. Consistently using design elements like characters, visuals, voices, and terminology throughout multiple touchpoints in the care pathway can provide children with a sense of familiarity. Providing such a sense of familiarity in an otherwise unfamiliar, stressful medical setting can give children and their parents a sense of comfort, continuity, and safety [25]. Using recognizable elements like characters can also provide staff with a starting point for conversation, helping them build rapport with the child.

### Objective

**Figure 1:**
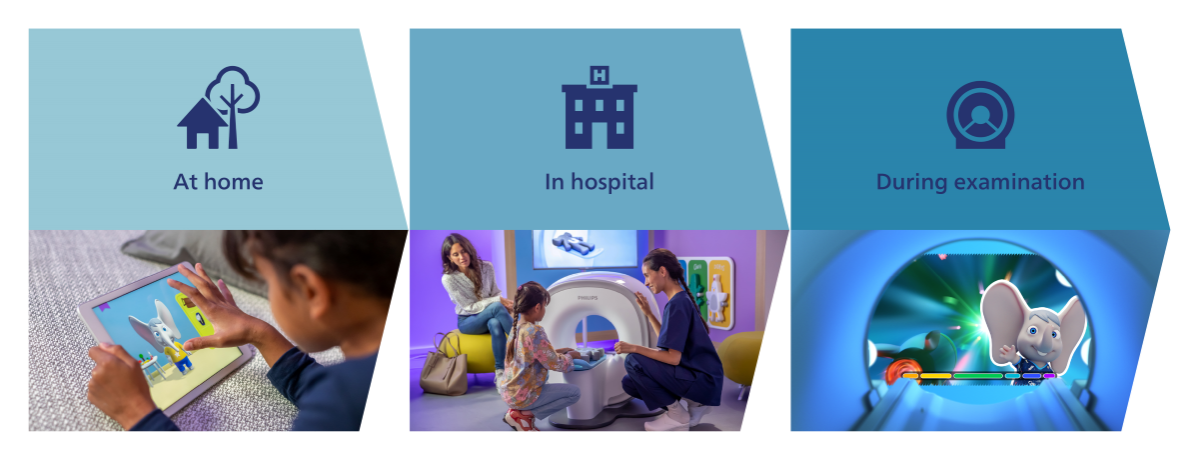
The complete Pediatric Coaching solution consists of the Scan Buddy app (left), enabling preparation at home days prior to the examination; the Kitten Scanner (middle), enabling preparation on-site moments before the examination; and pediatric In-bore (right), providing guidance during the examination and reusing familiar elements from the preparation.

To complete the full Pediatric Coaching solution (Figure 1), we aimed to design, develop, and test a solution that guides and engages children during their MRI examination. The goal was to develop a pediatric In-bore Experience solution (pediatric In-bore) as a variant of an existing In-bore Experience solution using design elements of our preparation solutions. By means of a usability study, we wanted to test whether children would like pediatric In-bore and understand the guidance it offered.

## Methods

### Design of Pediatric In-bore

#### Background

**Figure 2:**
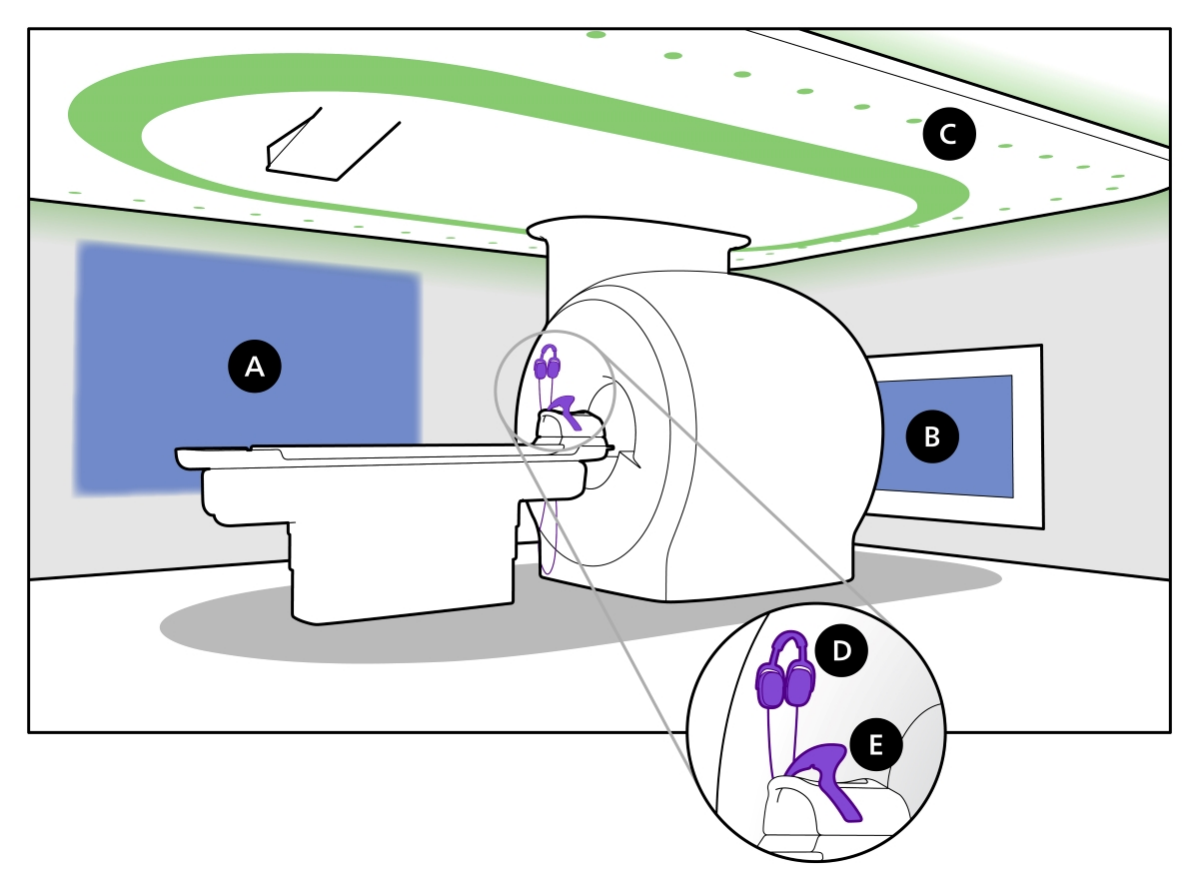
The hardware elements enabling In-bore Experience are (A) wall projection; (B) In-bore screen; (C) dynamic lighting that matches the colors of the visual content displayed on the wall projection or screen; (D) headphones; and (E) mirror.

Pediatric In-bore is a child-friendly adaptation of an existing In-bore Experience solution that engages and guides patients during their examination (see Figure 2 for an overview of its hardware elements). Supporting patients in the MRI room is important, as patients’ physiological anxiety levels are relatively high at the beginning of an MRI examination, with a peak when patients enter the bore [26]. Providing patients with information during the examination (eg, to inform them about how long the examination lasts), is associated with lower cortisol levels (an important indicator of physiological stress; [27]).

With In-bore Experience, patients can look outside the bore during the MRI examination using a mirror. In this mirror, they can see a screen showing audiovisual content, such as a movie. Patients wear headphones to hear the music or audio accompanying the content displayed on the screen. They can also use these headphones to communicate with staff and to hear automated voice messages. These automated messages (“AutoVoice”) instruct patients about when to hold still because the table moves and how to breathe during a breath-hold scan. They also inform patients about the duration of the upcoming scan (eg, “The next scan will last for three minutes”). Through a connection with the MRI scanner (“In-bore Connect”), information about the progress of the examination and breath holds can be shown as overlays on the audiovisual content.

For pediatric In-bore, we adapted three distinct elements of the In-bore Experience solution: the audiovisual themes, AutoVoice, and In-bore Connect. Below, we describe the design rationale for the pediatric version of each of these elements.

#### Themes

Themes consist of a video with sounds and music for the wall projection and In-bore screen, and associated lighting patterns for the dynamic lightning (Figure 2). There are themes for adults, as well as children, and patients can choose which theme they want to watch.

To complement the Pediatric Coaching solution, new videos were created featuring three characters: Ollie the Elephant, Chris the Crocodile, and Doris the Chicken. Children have been familiarized with these characters with the Kitten Scanner and (for Ollie) with the Scan Buddy app. Each character has his or her own story (Multimedia Appendix 1). The goal of themes is to engage children and prevent boredom and as such support them with lying still. The themes were designed with a storyline (eg, “Ollie’s space journey”) that consists of several substories (eg, “Ollie visits an underwater planet” and “Ollie visits a mushroom planet”) separated by a repetitive scene (eg, “Ollie in her rocket”). This setup was chosen because the length of an MRI examination can differ considerably; the modular setup ensures that children do not miss an exciting part of a story, because the examination finished midway.

Moreover, given that for some head and brain scans, even minor head motion can negatively affect image quality [28], the themes were designed with one central focus. Peripheral cues can draw attention, involuntarily directing people’s gaze toward the cue [29]. Some research states that such reflexive eye movements can cause motion artifacts [30]. As such, themes that avoid inducing many eye movements are preferable to themes that may induce a lot of eye movement.

#### AutoVoice

##### Background

Pediatric AutoVoice is a version of AutoVoice redesigned to provide a consistent experience throughout the Pediatric Coaching solution. The existing “adult” AutoVoice was transformed into a pediatric version tailored to the needs of young children by making several changes elaborated below.

##### Voice

For all 15 languages in which pediatric AutoVoice was created, we worked with the same voice actors as in the Scan Buddy app and Kitten Scanner. This was done to ensure that children hear the same voice at home, in the hospital, and during the MRI scanner. While adult AutoVoice has a practical, professional tone of voice, pediatric AutoVoice has an encouraging, lively tone of voice, using more inflection.

##### Language Use

The language used in pediatric AutoVoice was tailored to young children by:

- Using informal language for those languages that have a distinction between the formal and informal language (eg, “Sie” or “du” in German; “u” or “jij” in Dutch).
- Using the terminology used throughout the Pediatric Coaching solution, which is a child-friendly version of the terminology used for adults. For example, where adult AutoVoice uses terminology such as “table” and “scan,” pediatric AutoVoice uses terminology such as “bed” and “picture.”
- Using generic time indicators to indicate the duration of the next scan. For example, when adult AutoVoice says “The next scan will last for half a minute,” pediatric AutoVoice says “The next picture will be really quick.”

The announcements of scan duration were adapted because research shows that children younger than 8 years of age typically do not have an explicit sense of time that can be measured in concrete time units such as minutes or hours [31]. This is in line with Piaget’s [32] classic theory, which posits that children start to understand abstract concepts and symbolic representations such as time around age 7-8 years, when they move into the concrete-operational stage of development.

##### Timing

Providing automated breathing instructions is an important feature of adult AutoVoice. During a so-called breath-hold scan, the patient is instructed how to breathe and when to hold, most commonly with the instructions “Breathe in ... breathe out ... and hold your breath,” after which the scanner starts acquiring data. When the acquisition is finalized, the patient is told “You may breathe again.”

On average, children have higher breathing rates than adults [31]; as such, the AutoVoice timing was adjusted to better match children’s natural breathing patterns. In adult AutoVoice, on average, patients are instructed to breathe in and breathe out in approximately 2.5 seconds each (the exact length can differ across languages). This resembles a breathing frequency of 12 breaths per minute (BPM), which is at the low end of the natural breathing range spectrum for adults at rest.

For pediatric AutoVoice, we ensured that instructions had the same timing for all languages and shortened them to accommodate children’s natural breathing rates. In particular, the “Breathe in ...” and “breathe out ...” instructions were timed 2 seconds apart, in accordance with a breathing frequency of 15 BPM. With these instructions, we enable children to deeply breathe in a rhythm that matches their age, as 15 BPM is at the low end of the natural breathing range spectrum for young children at rest [33].

#### In-bore Connect

##### Background

In-bore Connect enables real-time visual patient guidance through a link with the MRI scanner. From the information received in real-time from the scanner about breath-hold scans and scan progress, overlays are constructed that are superimposed on the content displayed on the screen.

##### Breath-Hold Guidance (Visual)

When a breath-hold scan is coming up, AutoVoice informs the patient that the next scan is a breath-hold scan. To capture the patient’s attention, the content is dimmed, the progress bar hidden, and a “breathe” icon is displayed in the center of the screen. The moment the patient is instructed to hold their breathing, the icon changes and the circle that surrounds it is progressively filled to indicate the remaining time (Multimedia Appendix 2).

For pediatric In-bore Connect, the visuals of the breath-hold guidance were redesigned to have a more familiar and playful component. Upon announcing the breath-hold scan, the pediatric “breathe” icon is accompanied by Ollie, who encouragingly points to it to support the child to pay attention to what is coming next. The moment the child is instructed to hold their breath, the icon changes and the circle is animated to indicate the remaining time. Upon completing the breath hold, the icon changes back to the “breathe” icon and stars appear as a small reward for completing the breath hold (Multimedia Appendix 3).

##### Progress Bar

During the MRI examination, In-bore Connect enables the overlay of a progress bar at the bottom of the screen on top of the content shown. The progress bar indicates the total number of scans, their length, and the examination progress (Multimedia Appendix 4).

For pediatric In-bore, the progress bar was redesigned in such a way that it appeals to children and has the same familiar look and feel as the progress bar introduced in the Scan Buddy app and the Kitten Scanner (Multimedia Appendix 5).

##### Animated Ollie

Next to redesigning the breath-hold guidance and progress bar overlays, pediatric In-bore Connect introduces an animated Ollie as the third type of overlay. This animated Ollie is visible at the start and the end of the examination and displays various gestures like waving to the patient (Multimedia Appendix 6).

The goal of showing Ollie at the start of the examination is to actively engage and distract young children when they lie down on the table and the table slides into the MRI scanner, as this is a moment of high anxiety for patients [26]. As soon as the first scan starts, Ollie disappears and the progress bar starts filling, informing the child about the progress of the examination. When all the scans are finished and the progress bar has completely filled, Ollie pops up again. In this way, Ollie creates a small celebratory moment when the examination has been completed. She also bridges the time between finishing the last scan, and the operator coming into the room and moving the child out of the scanner.

### Pediatric In-bore Usability Study

#### Study Design

To investigate whether children would like and understand pediatric In-bore, we conducted a usability study with healthy participants. During a mock examination, participants were exposed to the different elements of pediatric In-bore: a pediatric theme, pediatric AutoVoice (in their native language), and the three overlays of pediatric In-bore Connect.

**Figure 3:**
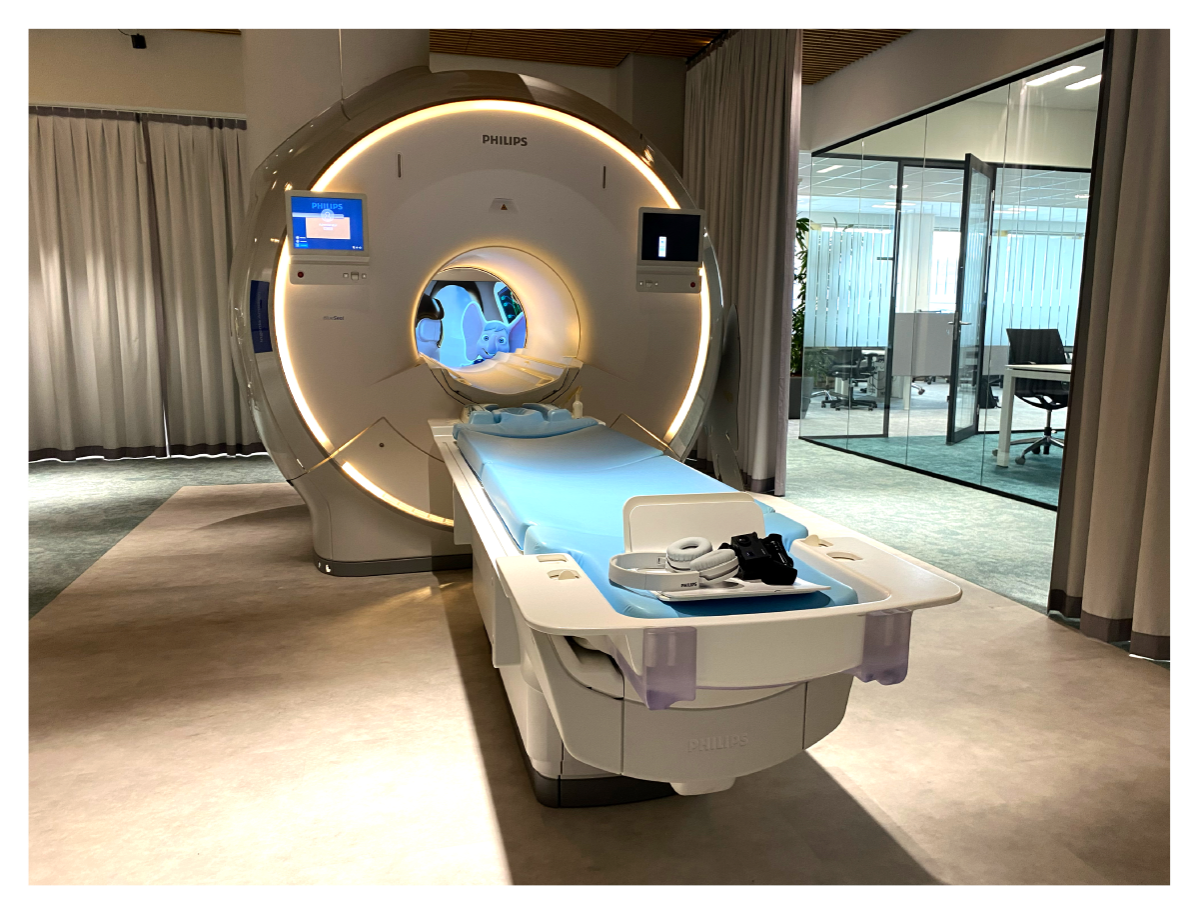
Picture of the mock MRI scanner in the usability laboratory, where the ~10-minute mock examination with the healthy participants (N=10; aged 5-10 years) of the pediatric In-bore usability study took place. MRI: magnetic resonance imaging.

The mock examination was conducted in a usability laboratory featuring a mock scanner that looks exactly like a functional MRI scanner (Figure 3). To simulate the full pediatric In-bore Experience during a real MRI examination, all the visuals (theme, as well as overlays) and sounds (MRI scanner sounds, as well as AutoVoice and theme sounds) were prerecorded and assembled into one video. The video was displayed on a screen behind the bore, which children could see and hear using an MRI mirror and headphones. After the mock examination, the children participated in a guided interview.

Each study session was facilitated by two researchers, one led the session and one observed and logged participants’ responses. Sessions were recorded to help code participants’ behavior inside the bore.

The study was conducted over the course of 4 weeks from mid-September to mid-October 2022 and lasted a maximum of 1 hour per participant.

#### Participants

Ten participants were recruited by an external recruitment agency that specializes in recruiting participants for qualitative research studies. They recruited the participants from their existing panel. Recruitment criteria were healthy children between the ages of 4 and 10 years, and speaking native English or Dutch. Participation was only allowed if the child was accompanied by a parent or legal guardian. Parents received a small compensation from the recruitment agency in the form of voucher points, while we gave the children a small gift package related to the Pediatric Coaching solution (small elephant snuggle, stickers, magnets, and a coloring book) after participation.

#### Ethical Considerations

Ethical approval for the study and all required documents, including the information letter, informed consent (with a section on privacy and confidentiality), and interview guide, were obtained from the Philips Internal Committee for Biomedical Ethics (study ICBE-S-000879), ensuring the study was conducted in accordance with the Declaration of Helsinki. The recruitment agency sent the information letter and informed consent to parents as part of recruitment, and consent was obtained prior to the study. During the introduction of the study on-site, child assent was also obtained by the researchers. Raw data such as video files and paper forms with identifying information were deleted or otherwise destroyed after extracting the relevant information and digitizing the data without identifying information other than gender, age, and participation number.

#### Outcomes

The main goal of the study was to investigate to what extent pediatric In-bore was liked and the guidance it provides understood by young children. As a secondary outcome, we gauged the ability of the child to lie still during the examination and to follow the breath-hold instructions.

#### Study Procedure and Material

Study sessions all started with a general introduction of what an MRI machine is and what it is used for, followed by an explanation of the study procedure. After that, the informed consent was signed by the parent, and a walk-through of child assent was done.

The main part of the study consisted of a mock examination of approximately 10 minutes. First, the child was equipped with a small camera on their head to enable the observers to inspect the behavior of the child when lying in the MRI scanner. Next, they were asked to climb onto the MRI bed. They were asked to lie as still as they could, like children undergoing a real MRI, and to watch the video and follow any instructions that were provided in the video. Then, they were donned with headphones so they could hear the audio embedded in the study video. The mirror was positioned, after which the bed was moved into the bore. While lying in the bore, the child watched the study video mimicking an MRI examination containing 5 MRI scans including 1 breath-hold scan.

**Figure 4:**
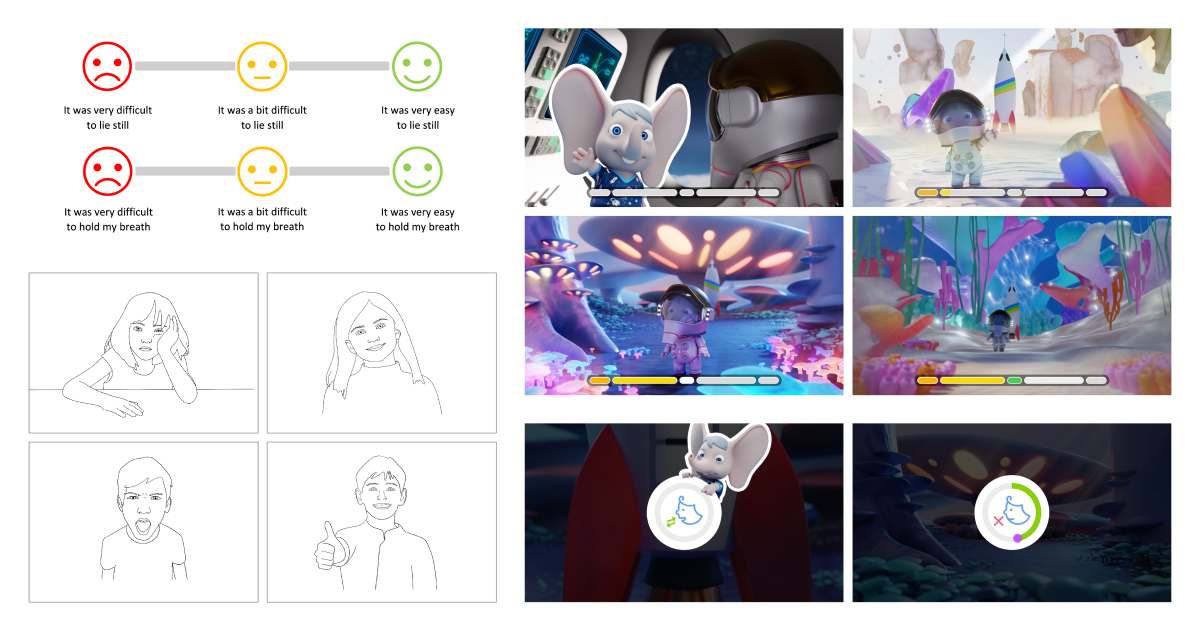
Examples of the rating scale and probing material (emotion line drawings, screenshots of the video) used during the ~35-minute guided interview with the healthy participants (N=10; aged 5-10 years) of the pediatric In-bore usability study.

Throughout the study, the Ollie theme was playing full screen, accompanied by the progress bar displaying the examination progress (Figure 4, top right). Pediatric AutoVoice announced the duration for each of the scans without breath holds and provided instructions throughout the breath-hold scan. The middle scan was the breath-hold scan containing 4 breath holds of varying lengths (4-10 s each), during which the breath-hold guidance was shown. During breath-hold guidance, the theme was dimmed and the progress bar was invisible (Figure 4, bottom right). At the start and the end of the study video, an Animated Ollie was shown (Figure 4, top middle).

The last part of the study was a guided interview to learn about the child’s experience during the mock examination and their opinion about the various elements of pediatric In-bore. This part lasted for approximately 35 minutes. To facilitate a smooth interview, screenshots of the study video were used to jog participants’ memory (Figure 4, right), and printed probes—line drawings of children with various emotions—were used to encourage talking and expression of feelings (Figure 4, bottom left). Three-point smiley rating scales were used to get an indication of how difficult or easy tasks were (Figure 4, top left).

#### Measures

To estimate how well the child executed the various parts of the mock examination (“Tasks”), children’s behavior was observed both directly, as well as during data analysis (using the video recording made inside the bore). For each task (see Table 1 for an overview of the tasks), it was coded if the child was able to perform the task “successfully (without any issues),” “successfully with minor issues,” or “unsuccessfully (with major issues)”.

**Table 1:** The tasks embedded in the ~10-minute mock examination of the pediatric In-bore usability study in chronological order, and the intended behavior of the healthy participants (N=10; aged 5-10 years) for each of them.

Tasks	Intended behavior
Lying down, going into the bore, and viewing animated Ollie	Going in without issues; watching the animation without movement
Lying still during the first two scans	Lying still without movement
Executing the breath holds during the middle, breath-hold scan	Correctly executing the four breath holds (after exhaling, holding their breath for the indicated duration)
Lying still during the last two scans, and viewing animated Ollie	Lying still without movement

For all tasks, small (localized) movements of the extremities (like wiggling toes or moving fingers) were coded as “minor issues,” as for the large majority of scans, these movements will not lead to images that are of nondiagnostic quality. Large body movements (shifting, rolling, arm gestures, attempting to get up) that are likely to have a major impact on the diagnostic quality of the scan were coded as “major issues.”

For the breath-hold task, task compliance was coded as “successful with minor issues” if the child conducted the breath holds, but (partially) did so in a wrong way (eg, after inhaling instead of exhaling). It was coded as “unsuccessful (with major issues)” if, for one or more of the breath holds, the instructions were observably not followed (no attempt to hold the breath or not able to maintain it).

The guided interview after the mock examination contained open questions and questions the child could answer by pointing to a position on a printed slider showing three emoticons (Figure 4, top left for an example). Besides general questions about the mock examination, specific questions were asked about the various elements of pediatric In-bore (see Multimedia Appendix 7 for a full overview of the self-reported outcome measures). For data analysis, the sentiment of the answers to the open questions was coded to a 3-point scale (negative, neutral, and positive). For example, if a child said “I love Ollie!” this answer would be coded as positive for likeability of Ollie. The slider position was coded as a 5-point scale.

## Results

### Participants

Ten healthy children (4 male and 6 female) aged 5 to 10 (mean age 7.6, SD 1.9) years, who had never undergone an MRI, participated in the study. All the participants underwent the mock examination and participated in the guided interview. Despite the supporting interview material, the two 5-year-old participants did not always seem to understand the question, nor were they always able to clearly communicate their opinions to the investigators, especially at the end of the interview. When possible, their answers were scored (this frequently resulted in a negative or neutral or did not notice score), while for some outcomes, no data are available at all (in these cases, N<10).

### Appreciation of Pediatric In-bore and its Elements

#### Overview

Table 2 provides the overview of the opinions voiced by children during the guided interview in the open questions. Table 3 provides the data from the slider scales.

**Table 2:** Self-reported outcomes of the healthy participants (N=10; aged 5-10 years) interpreted from the open questions asked during the ~35-minute guided interview of the pediatric In-bore usability study.

	Negative, n	Neutral, n	Positive, n	Did not notice, n
Overall examination experience (n=10)	0	2	8	N/A^a^
Likability of Ollie (n=10)	0	2	8	N/A
Likability of the Ollie theme (n=10)	0	2	8	N/A
Likability of the voice (n=10)	0	3	7	N/A
Understanding of verbal (scan duration) information (n=10)	1	1	7	1
Understanding of verbal breath-hold guidance (n=10)	0	1	9	N/A
Understanding of visual breath-hold guidance (n=10)	0	0	10	N/A
Understanding the progress bar (n=10)	0	0	8	2
Understanding the intent of animated Ollie at the start (n=10)	1	1	8	N/A
Understanding the intent of animated Ollie at the end (n=10)	0	0	8	2

^a^Not applicable.

**Table 3:** Self-reported outcomes of the healthy participants (N=10; aged 5-10 years) measured with sliders during the ~35-minute guided interview of the pediatric In-bore usability study.

	No, n	Not really, n	Do not know, n	Think so, n	Yes, n
Knowing when to lie still (n=9)	2	0	0	0	7
Knowing what to do (n=10)	1	0	1	0	8
Pediatric In-bore considered helpful (n=10)	1	0	1	2	6
Knowing when the first scan started (n=9)	2	0	0	0	7
Knowing when the last scan ended (n=8)	0	0	0	0	8

#### Ollie

Ollie and her theme were generally liked, with the same two participants (a 5-year-old and an 8-year-old) scoring neutral for both outcomes. The animated Ollie (Figure 4, top middle) was not clearly identifiable or memorable for all participants in comparison to the Ollie shown in the theme. When asking the participants about animated Ollie and what they thought Ollie was doing, they often started to explain the theme’s storyline. By explicating that we were interested in what they thought the animated Ollie was doing, the majority of participants (8/10, 80%) correctly indicated that Ollie was waving hi, or welcoming them at the start of the examination, and waving goodbye, or applauding them at the end; for example, a participant said:

He means like good job. I saw it because of the wink, because when you wink it often means you did a good job.

#### Progress Bar

A total of 8 (80%) out of 10 participants understood the progress bar and were able to explain it in their own words:

That was really nice. Because, when it was a short one, it has a short bar, and the long one that took a long time, there was a long one. And every time it made a different sound with the bar…It was really handy, because you sort of knew when you’d be done.

Both 5-year-old participants mentioned not seeing or noticing the progress bar, and they either did not answer, or answered negatively to the questions about whether they knew when the first scan started, or the last scan finished.

### Task Success

#### Overview

As a secondary outcome, task success was coded. One participant (a 5-year-old child) executed the mock examination with major issues on all tasks, while the rest of the 9 participants were able to perform most of the tasks without any issues or with minor issues (Table 4).

**Table 4:** Observed task success of the healthy participants (N=10; aged 5-10 years) during the ~10-minute mock examination of the pediatric In-bore usability study.

	Unsuccessful (with major issues), n	Successful with minor issues, n	Successful (without any issues), n
Lying down, going into the bore, and viewing animated Ollie (n=10)	1	0	9
Lying still during the first two scans (n=10)	1	4	5
Executing the breath holds during the breath-hold scan (n=10)	4	4	2
Lying still during the last two scans, and viewing animated Ollie (n=10)	1	3	6

Next to observing their behavior, we also asked children about their perceived ability to lie still and hold their breath (see Table 5). The participant who was unable to complete the tasks successfully (due to large body movements) indicated that she found it very difficult to lie still, and she failed to answer the question about holding one’s breath.

**Table 5:** Self-reported task success of the healthy participants (N=10; aged 5-10 years) during the ~10-minute mock examination of the pediatric In-bore usability study.

	Very difficult, n	Difficult, n	A bit difficult, n	Pretty easy, n	Very easy, n
Perceived ability to lie still (n=10)	1	0	0	3	6
Perceived ability to hold breath (n=9)	0	0	2	3	4

#### Breath-Hold Scan

Of all the tasks, correct execution of the 4 breath holds during the breath-hold scan was clearly the most difficult. Only 2 participants (a 6-year-old and a 9-year-old) perfectly held their breath after exhaling for all 4 breath holds. Four participants did successfully hold their breath during all breath holds, but not always in the exact way AutoVoice indicated, while 4 participants did not correctly hold their breath for 1 or 2 of the 4 breath holds.

The AutoVoice said “Breathe in ... breathe out and hold your breath” with the visual guidance supporting the transition from breathing to the breath hold (Multimedia Appendix 3). Objectively, we observed the children make solid attempts, but frequently they took a quick breath in before holding, instead of pausing breathing after exhaling as was instructed. We also noted that some children displayed a learning curve, taking a bigger breath when instructed to breathe in and out during the later breath holds compared to the earlier ones.

When discussing their performance, several children—including ones who paused their breathing at the correct moment—questioned the accuracy of the instructions. For example, one participant remarked:

Hold your breath, I did not get that completely. Because it was breathe in, breathe out, and hold your breath, but how is that possible? Because your breath is already gone!

Emotionally, the breath-hold scan was one of the most discussed and commented parts of the mock examination, with some participants expressing feelings of pride in being able to hold their breath and others elaborating on similar breath-holding experiences, for example, during swimming underwater.

## Discussion

### Principal Findings

We developed a pediatric In-bore Experience solution using design elements of our preparation solutions, thereby creating a holistic coaching solution to support patients at home, in the hospital, and during their MRI. The usability study demonstrated that overall, young children were positive about pediatric In-bore and indicated that it helped them during the examination. Ollie and the theme were liked, and pediatric AutoVoice and accompanying breath-hold guidance and progress bar visual overlays were generally liked and understood by most children. All but one child successfully completed the majority of the tasks embedded in the mock examination, that is, lying down on the bed, entering the bore, and lying still during the scans, and most participants successfully held their breath.

### Breath-Hold Scans

One of the most challenging aspects of the mock examination was the breath-hold scan, which is required for a small subset of all MRI examinations: namely, those of anatomies that are affected by breathing motion, such as cardiac scans and liver scans. Breath-hold scans make up a small percentage of all scans since the majority of pediatric examinations are head scans or brain scans (see eg, [34]). Nevertheless, we wanted to provide some support for this type of scan because it is known to be a challenging examination for (pediatric) patients.

In this usability study, we instructed participants to hold their breath after exhaling, as this is the common approach in clinical practice. The reason for this is that holding one’s breath after exhaling is—when executed correctly—more reproducible than holding it after inhaling, thus resulting in better image quality (see eg, [35]). However, since holding one’s breath is familiar to children after inhaling (this is what many children learn during swimming lessons), the counterintuitive instructions of holding one’s breath after exhaling can be difficult to adhere to. This is consistent with anecdotal evidence indicating that experienced MRI technologists often opt to conduct breath-hold scans after inhaling for young patients.

Familiarizing the child with the instructions and expected behavior during breath-hold scans and practicing breath holds before their examination is also likely to improve their breath-hold execution [36,37]. Integrating such a feature in patient preparation solutions is therefore recommended based on the results of our usability study.

### Technical Advancements that Support Awake Scans

In addition to improved preparation and guidance, there are ongoing technical advancements in MRI that are making it easier to scan young children awake. For example, breath holds are becoming shorter [38], and for an increasing number of breath-hold scans, free breathing alternatives are being developed, yielding acceptable diagnostic quality that is, in many cases, comparable to the diagnostic quality of breath-hold scans [39-42]. Moreover, emerging artificial intelligence–based techniques provide accelerated acquisition and motion robustness for MRI [43]. The technical advancements that shorten MRI scans and make them more resilient to motion, coupled with effective patient preparation and guidance solutions, create a powerful combination to support the minimization of sedation or anesthesia use in pediatric MRI.

### Limitations

In this study, one child found it very difficult to lie still in the scanner and to follow the instructions. This could be partially because the participants were not prepared for the mock examination. Pediatric In-bore is designed to be part of a broader solution to support children in their MRI journey, consisting of preparation at home (Scan Buddy app), in the hospital (Kitten Scanner), and guidance during the examination (pediatric In-bore), but this usability study was limited in that it only included the last part of the solution. More research is needed to investigate the impact of the full Pediatric Coaching solution on patient experience and the clinical workflow.

Another limitation of this study was that only children between the ages of 5 and 10 years were included, while pediatric In-bore may also be valuable for younger and older children. We did not find marked age differences in liking pediatric In-bore or Ollie, but this could be due to the small sample size: we only included 10 participants. The generalizability of our findings is particularly limited because there are large intraindividual differences between children in terms of, for example, their developmental level, preferences, coping styles, and ability to understand instructions. This diversity between children is even more pronounced within a hospital setting, in which children’s chronological age may not necessarily align with their developmental level (eg, in the case of children with cognitive impairments [44]). Conceivably, in clinical practice, there may be some teenagers or even adults (eg, those with cognitive impairment) who prefer pediatric In-bore over the adult In-bore Experience.

In the end, any patient preparation or guidance will be most effective if a certified child life specialist, play therapist, or other staff member (such as an MRI technologist or the child’s referring physician) can tailor it to patients [44]. For stronger conclusions on the liking and acceptance of pediatric In-bore in the pediatric patient population, more research is needed in a larger and more diverse sample of pediatric patients.

### Conclusions

The pediatric In-bore Experience solution includes child-friendly themes, automated voice instructions tailored to children, and three types of informative and engaging overlays; all were designed to support and guide young children during their MRI. Pediatric In-bore seems a promising approach to engage and guide young children during awake MRI examinations, as this usability study showed that it was liked and understood by most of the participants aged 5-10 years.

Although patient preparation and guidance may strongly reduce the need for anesthesia or sedation in pediatric MRI, we expect that there will always be children for whom it will be challenging to keep still during the scan. This is in line with previous studies that suggest that most, but not all children are able to undergo a successful awake examination (even if they have been prepared for their examination). For example, both Geuens et al [24] and Runge et al [21] report a success rate of 95% (in these studies, 5% of children could not successfully be scanned awake). If a child is scheduled for an awake examination but ends up requiring anesthesia, this can be challenging for hospitals, as well as for children and their families.

Pediatric In-bore complements the Pediatric Coaching solution, which further consists of the Scan Buddy app (to prepare children at home [23]) and the Kitten Scanner (to prepare children in the hospital [24]). More research is needed to investigate the impact of the full Pediatric Coaching solution on patient experience and the clinical workflow. Given the ongoing debate on the safety of repeated use of anesthesia in young children, it is crucial to find solutions to scan children awake that are safe, effective, and child-friendly. Holistic solutions that can prepare children for their examinations and guide them throughout the process can provide hospitals with new tools to reduce the need for anesthesia or sedation in pediatric MRI, although more research is needed to fully understand how such solutions affect anesthesia and sedation rates in a hospital setting.

## Data Availability

The data sets generated during or analyzed during this study are not publicly available due to the company’s privacy policy.

## Appendix

Multimedia Appendix 1 Screenshots of the three pediatric themes created for pediatric In-bore Experience featuring the characters of the Pediatric Coaching solution: Ollie the elephant (top); Chris the crocodile (middle); and Doris the chicken (bottom).

Multimedia Appendix 2 Screenshots of the visual breath-hold guidance provided by the regular In-bore Experience solution through In-bore Connect: alternately displaying a "breathe" icon (top) and a "hold breath" icon + animated progress circle (bottom).

Multimedia Appendix 3 Screenshots of the visual breath-hold guidance provided by pediatric In-bore through In-bore Connect: breath-hold scan announcement displaying the "breathe" icon and Ollie (top); breath-hold progress displaying the "hold breath" icon and the progress (middle); and breath hold completed displaying the "breathe" icon and celebrative stars (bottom).

Multimedia Appendix 4 Example progress bar provided by the regular In-bore Experience solution through In-bore Connect that shows the following: the first two scans have been completed, the third scan is ongoing, and three scans are remaining.

Multimedia Appendix 5 Illustration of the colorful examination progress bar as a recurring design element in the Pediatric Coaching solution: in the Scan Buddy app (top); the Kitten Scanner (middle); and pediatric In-bore (bottom).

Multimedia Appendix 6 Screenshots of pediatric In-bore displaying that at the start (top) and end (bottom) of the MRI examination Ollie the elephant actively welcomes and says goodbye to the child. MRI: magnetic resonance imaging.

Multimedia Appendix 7 Structured overview per element of pediatric In-bore of the outcome measures and their question type that were embedded in the ~35-minute guided interview with the healthy participants (N=10; aged 5-10 years) of the pediatric In-bore usability study.

## Abbreviations

BPM: breaths per minute
MRI: magnetic resonance imaging
