# Supplementary figures and images for: Improving Pediatric Patients’ Magnetic Resonance Imaging Experience With an In-Bore Solution: Design and Usability Study
Source: JMIR Serious Games. 2025 Feb 13;13:e55720. doi: 10.2196/55720 (PMC11888108; doi:10.2196/55720)

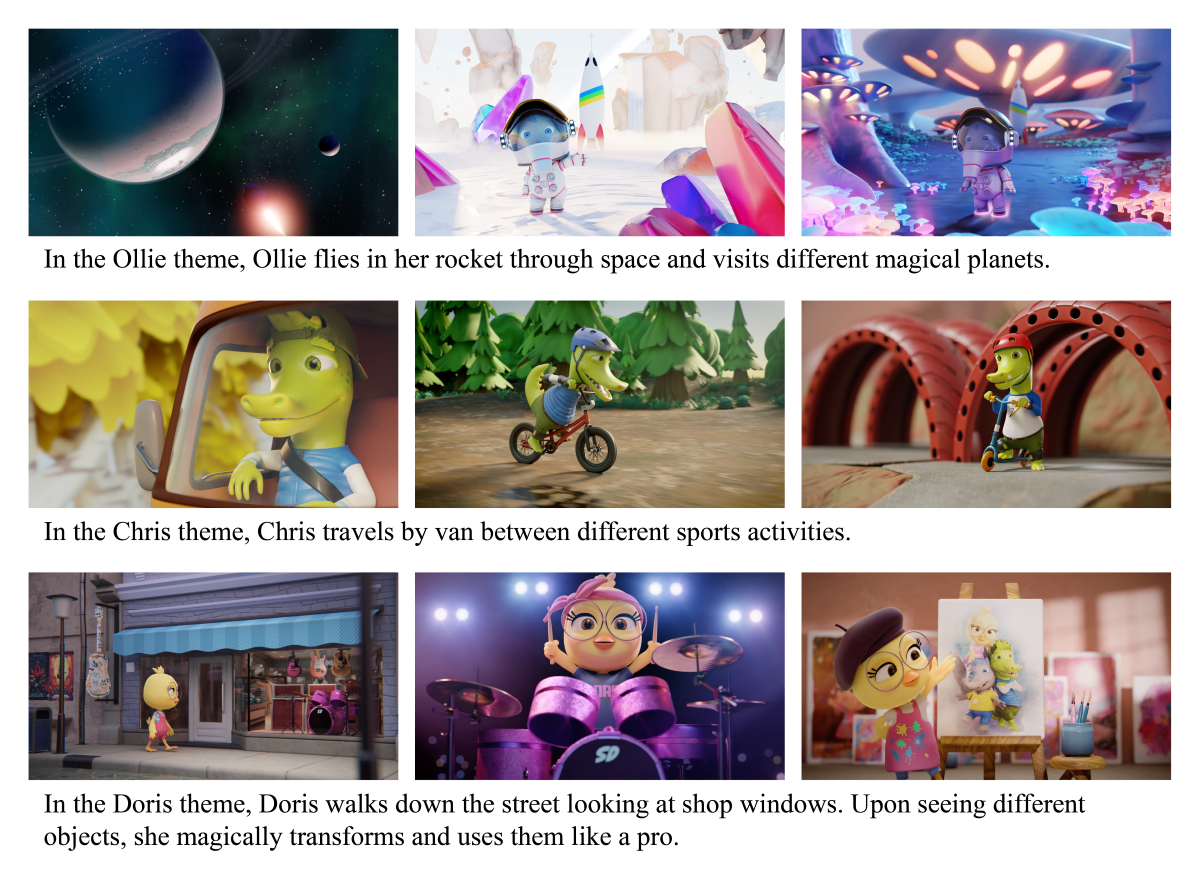

Supplement: Multimedia Appendix 1 [file games_v13i1e55720_app1.png]

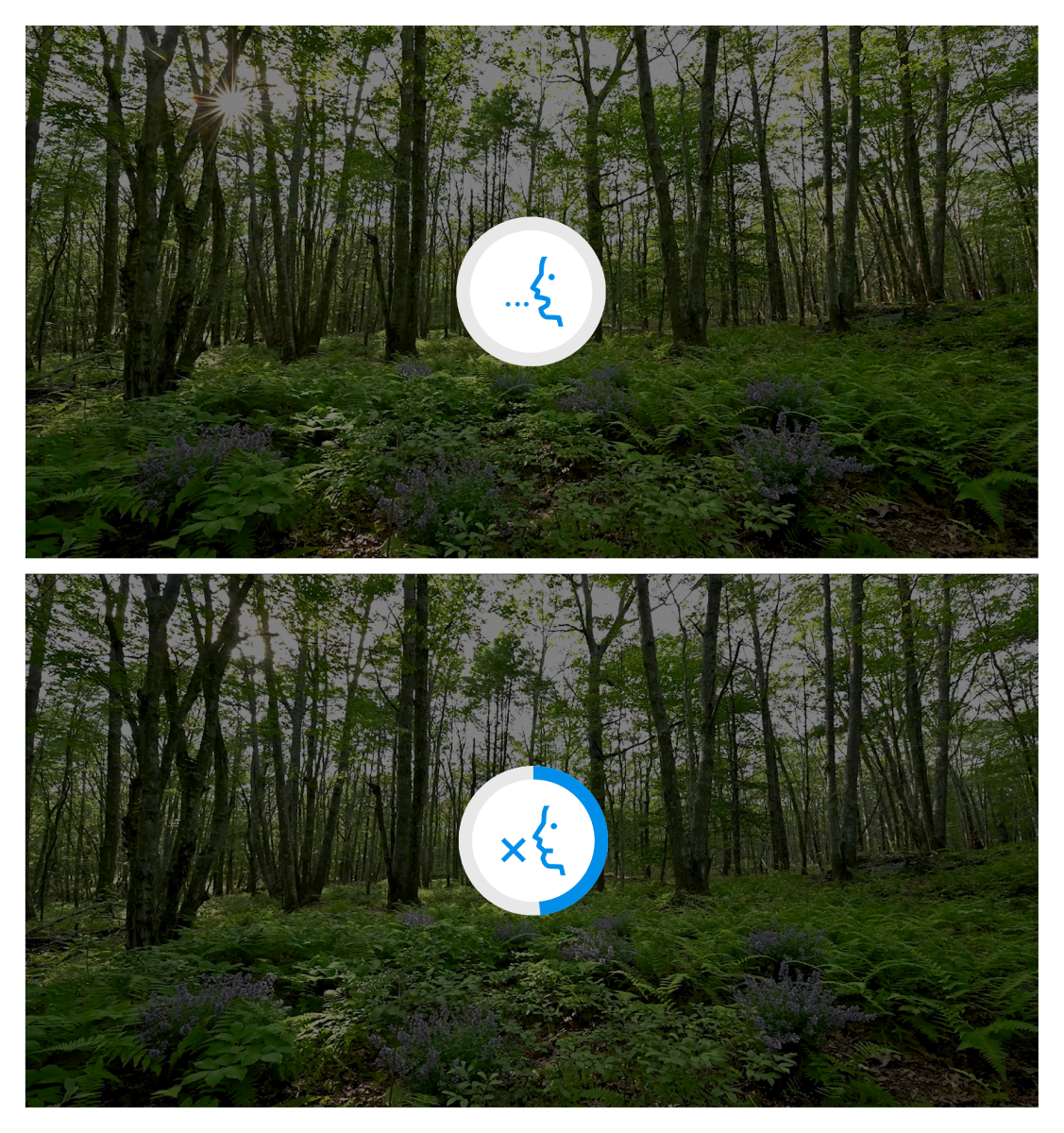

Supplement: Multimedia Appendix 2 [file games_v13i1e55720_app2.png]

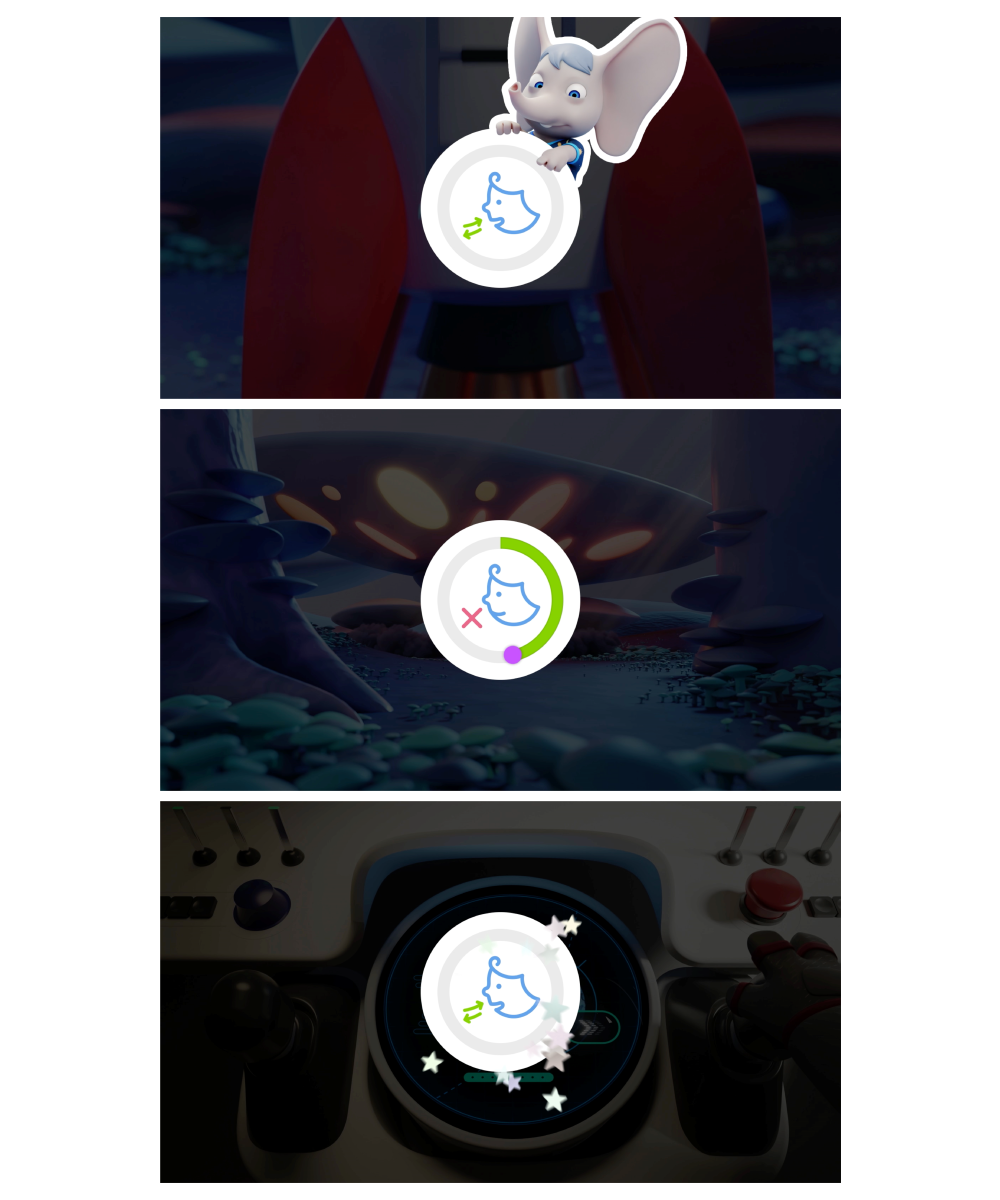

Supplement: Multimedia Appendix 3 [file games_v13i1e55720_app3.png]

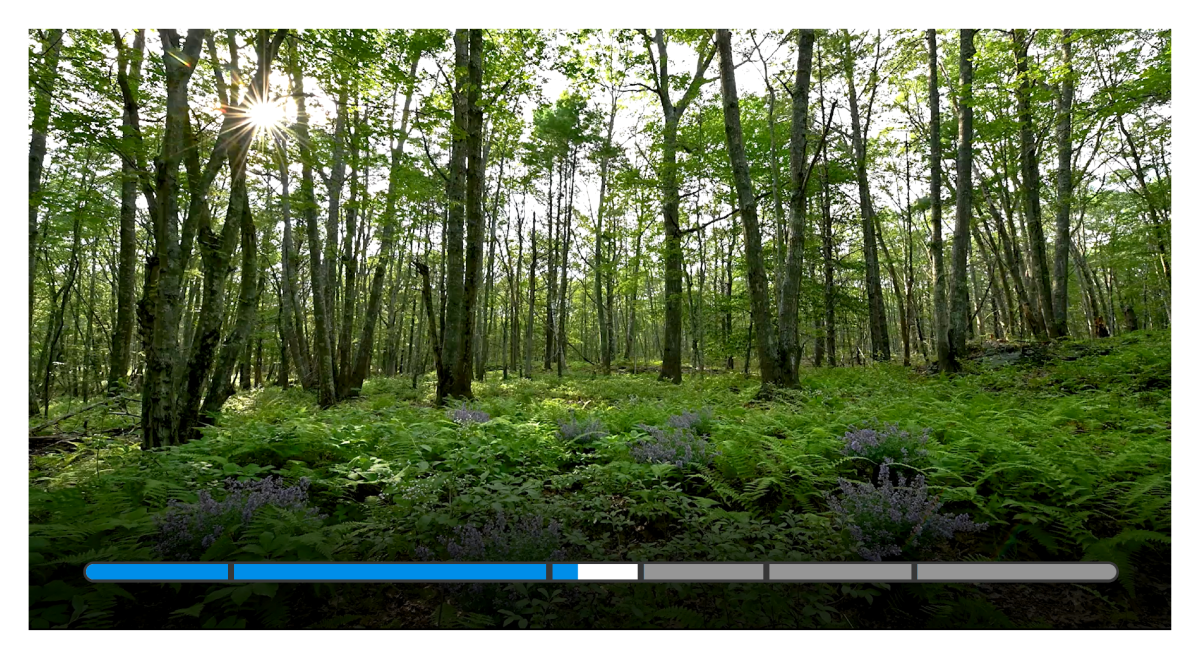

Supplement: Multimedia Appendix 4 [file games_v13i1e55720_app4.png]

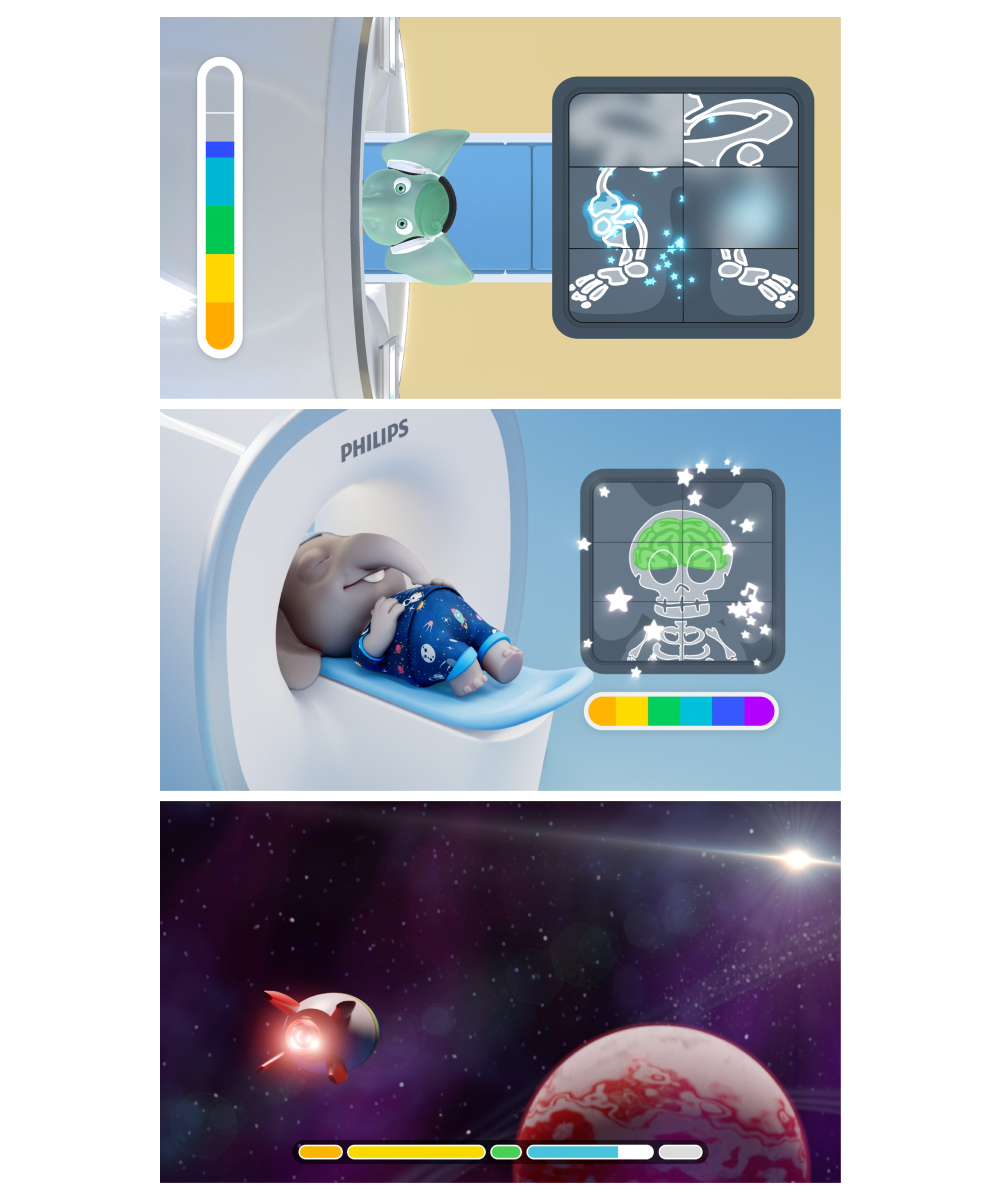

Supplement: Multimedia Appendix 5 [file games_v13i1e55720_app5.png]

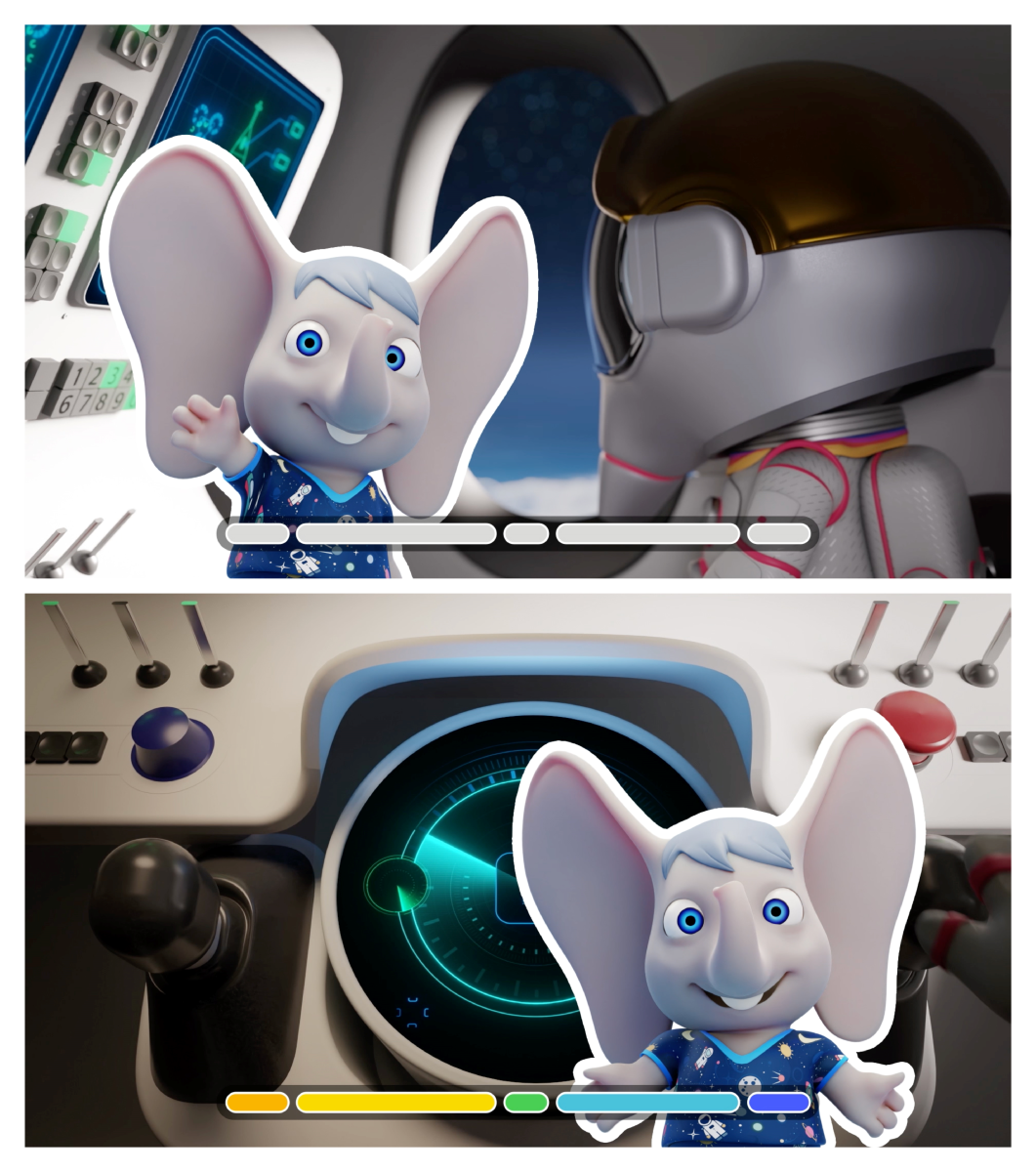

Supplement: Multimedia Appendix 6 [file games_v13i1e55720_app6.png]
