# Supplementary material for: Improving Pediatric Patients’ Magnetic Resonance Imaging Experience With an In-Bore Solution: Design and Usability Study
Source: JMIR Serious Games. 2025 Feb 13;13:e55720. doi: 10.2196/55720 (PMC11888108; doi:10.2196/55720)
Supplement: Multimedia Appendix 7 [file games_v13i1e55720_app7.docx]

|  | Outcomes | Question type |
| --- | --- | --- |
|  |  |  |
| **Complete pediatric In-bore** |  |  |
|  | Overall exam experience | Open (3-point) |
|  | Knowing when to lie still | Slider (5-point) |
|  | Perceived ability to lie still | Slider (5-point) |
|  | Knowing what to do | Slider (5-point) |
|  | Pediatric In-bore considered helpful | Slider (5-point) |
|  |  |  |
| **Ollie theme** |  |  |
|  | Likability of Ollie | Open (3-point) |
|  | Likability of the Ollie Theme | Open (3-point) |
| **Pediatric AutoVoice** |  |  |
|  | Likability of the voice | Open (3-point) |
|  | Understanding of verbal (scan duration) information | Open (3-point) |
|  | Understanding of verbal breath-hold guidance | Open (3-point) |
|  | Perceived ability to hold breath | Slider (5-point) |
| **Breath-hold guidance (visuals)** |  |  |
|  | Understanding of visual breath-hold guidance | Open (3-point) |
|  | (*copy*) Perceived ability to hold breath | Slider (5-point) |
| **Progress bar** |  |  |
|  | Understanding the progress bar | Open (3-point) |
|  | Knowing when the first scan started | Slider (5-point) |
|  | Knowing when the last scan ended | Slider (5-point) |
| **Animated Ollie** |  |  |
|  | (*copy*) Likability of Ollie | Open (3-point) |
|  | Understanding the intent of animated Ollie at the start | Open (3-point) |
|  | Understanding the intent of animated Ollie at the end | Open (3-point) |
